# Trigger-Free and Low-Cross-Sensitivity Displacement Sensing System Using a Wavelength-Swept Laser and a Cascaded Balloon-like Interferometer

**DOI:** 10.3390/s25030750

**Published:** 2025-01-26

**Authors:** Jianming Zhou, Jinying Fan, Junkai Zhang, Jianping Yao, Jiejun Zhang

**Affiliations:** 1 Guangdong Provincial Key Laboratory of Optical Fiber Sensing and Communication, Institute of Photonics Technology, Jinan University, Guangzhou 510632, China; zhoujianming@stu2020.jnu.edu.cn (J.Z.);; 2 College of Physics & Optoelectronic Engineering, Jinan University, Guangzhou 510632, China

**Keywords:** microwave photonic sensor, optical fiber sensor, wavelength-swept laser, displacement measurement

## Abstract

A wavelength-swept laser (WSL) demodulation system offers a unique time-domain analysis solution for high-sensitivity optical fiber sensors, providing a high-resolution and high-speed method compared to optical spectrum analysis. However, most traditional WSL-demodulated sensing systems require a synchronous trigger signal or an additional optical dispersion link for sensing analysis and typically use a fiber Bragg grating (FBG) as the sensing unit, which limits displacement sensitivity and increases fabrication costs. We present a novel displacement sensing system that combines a trigger-free WSL demodulation method with a cascaded balloon-like interferometer, featuring a simple structure, high sensitivity, and low temperature cross-sensitivity. The sensor is implemented by bending a short length of single-mode fiber with an optimal radius of around 4 mm to excite cladding modes, which form an interference spectral response with the core mode. Experimental findings reveal that the system achieves a high sensitivity of 397.6 pm/μm for displacement variation, corresponding to 19.88 ms/μm when demodulated using a WSL with a sweeping speed of 20 nm/s. At the same time, the temperature cross-sensitivity is as low as 5 pm/°C or 0.25 ms/°C, making it a strong candidate for displacement sensing in harsh environments with significant temperature interference.

## 1. Introduction

High-sensitivity displacement sensors hold significant research value in precision measurement and can be used in astronautical technology [1], continuous respiratory monitoring [2], and the manufacturing industry [3]. Optical fiber sensor technology uses optical fibers as a medium, leveraging the high speed, large bandwidth, and multiplexing capability of modern interconnected networks, making it applicable to many sensing scenarios [4]. Recently, researchers have reported micro-displacement sensors utilizing a balloon-like optical fiber structure and intermodal interference, highlighting their simplicity, low cost, and resistance to electromagnetic interference [5,6]. Traditional bent fiber sensing systems typically employ amplified spontaneous emission (ASE) as the broadband optical source and an optical spectrum analyzer (OSA) as the interrogator [7]. However, this demodulation system for bent fiber sensors is inadequate for high-resolution and high-speed measurements, and temperature fluctuations significantly impact sensing accuracy for micro-displacement tests [8]. Therefore, improved demodulation technology that offers high resolution, high sensitivity, and low cross-sensitivity is highly desired.

Microwave photonic sensing [9,10] utilizes microwave photonic techniques to achieve high-speed and high-resolution demodulation for micro-displacement [11], pressure [12], temperature [13], and refractive index [14]. Currently, demodulation approaches for microwave photonic sensing systems include those based on microwave photonic filters (MPFs) [15], optoelectronic oscillators (OEOs) [16], micro resonators (MRs) [17], and wavelength-swept lasers (WSLs) [18]. Among these technologies, MPF-based and OEO-based sensor demodulation can only demodulate changes in narrow-band or specific periodic spectra and is unsuitable for the demodulation of optical sensors with broadband optical spectral characteristics. For the demodulation of bent fiber sensors, a wavelength-swept laser (WSL), in conjunction with a high-speed photodetector, enables rapid measurement of the spectral response of the optical sensor, thereby achieving high-speed, high-sensitivity, and high-resolution demodulation [19,20,21].

The traditional measurement of the WSL-based demodulation system involves a laser source that can generate an extremely precise synchronization signal, requiring a synchronous trigger signal or an additional optical link as a reference point to achieve the sensing data analysis [22,23]. The WSL-based scheme commonly has two electrical-matched links, resulting in high system complexity and weak anti-interference capability. To simplify this setup, researchers have replaced electrical links with one optical link incorporating a dispersion module as a time mark [24,25]. In these approaches, the pulse from the WSL is divided into two parts by an optical circulator. One part directly passes through the sensor and photodetector for displacement measurement, while the other serves as a clock signal, which must be matched to the primary path via the dispersion module [26]. Although these methods can demodulate the shift of most bent fiber sensors, the requirement for two paths and synchronization still introduces complexity issues, making the system significantly more complex and expensive. Therefore, developing a high-sensitivity micro-displacement WSL sensing system with a simpler structure and lower cost is of great significance.

In this paper, we propose a novel high-sensitivity displacement sensing system that utilizes a trigger-free WSL link combined with a cascaded balloon-like bent fiber sensor. The system employs two bent single-mode fibers to form interferometers based on intermodal interference, with one acting as the sensing unit and the other as the reference. Simulations were conducted to assess the impact of different bending radii on transmission, and displacement measurement experiments were performed. When the displacement of the balloon-like fiber sensor changes, the optical filter responses change inversely, resulting in a shift of the waveform in the time domain. The experimental results demonstrate that the system achieves a displacement sensitivity of 19.88 ms/µm and an exceptionally low temperature sensitivity of 0.25 ms/°C at a sweeping speed of 20 nm/s, corresponding to a high displacement sensitivity of 397.6 pm/µm and a low cross-sensitivity of 5 pm/°C. This system not only mitigates the impacts of environmental disturbances but also reduces the overall cost of the WSL system due to the single-link design incorporating an optical mark. Moreover, this method provides a high-sensitivity displacement sensing demodulation system with low cross-sensitivity, and the cascaded balloon-like interferometer can be extended to other parameter measurements.

## 2. Principle

**Figure 1:**
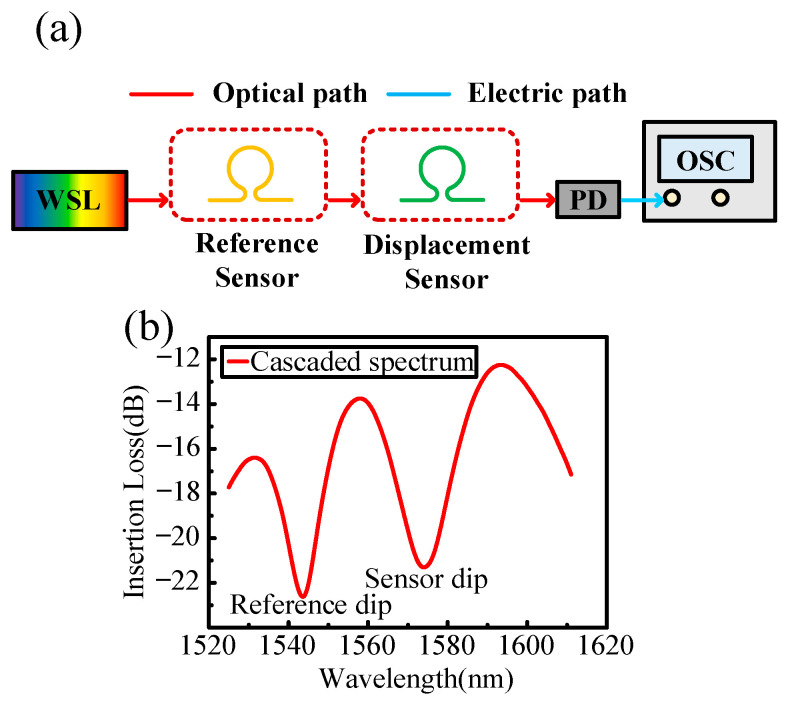
The schematic of a wavelength-swept laser sensing system with a reference sensor and a displacement sensor. (**a**) Structure of the entire system. (**b**) Transmission spectrum of the cascaded balloon-like bent fiber sensors. WSL, wavelength-swept laser; PD, photodetector; OSC, oscilloscope.

Figure 1a shows the schematics of the WSL sensing system. The WSL pulse passes through a reference sensor and the displacement sensor and is then detected by a photodetector (PD) and sampled by an oscilloscope (OSC). The reference sensor and displacement sensor are fabricated by bending a short-length single-mode optical fiber to form a balloon-like structure. With a small bending radius, the propagating core mode couples to cladding modes, which then interfere with the residual core mode, generating an interference spectrum. As shown in Figure 1b, the spectrum of the cascaded balloon-like bent fiber sensors reveals two distinct interference dips in the range of 1520–1620 nm. Since the optical trigger unit remains stationary, its interference dip remains unchanged, providing a stable signal reference point. When a displacement is applied to a balloon-like bent fiber sensor, the bending radius will be changed, resulting in a shift of interference dip. Displacement sensing data can be obtained by measuring the differences in sweeping times between the two interference dips

**Figure 2:**
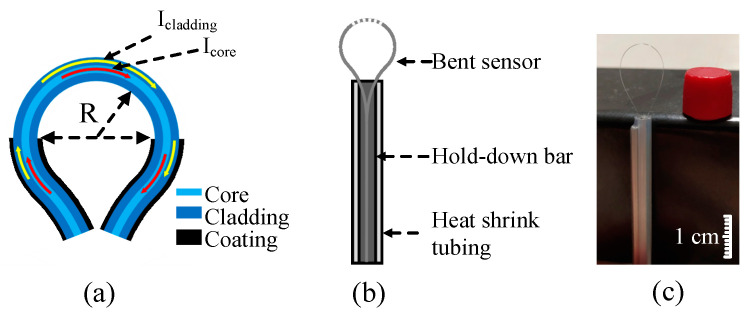
The structure of the balloon-like bent fiber sensor and photograph. (**a**) Schematic diagram of the coupling between core and cladding modes. (**b**) Structure of the balloon-like bent fiber sensor. (**c**) Photograph of real product. R, radius.

Figure 2 illustrates the structural and fabrication details of the balloon-like interferometer and the real sensor used in our experiment. In Figure 2a, the displacement sensor consists of a bare standard single-mode fiber (SMF) with a fixed bending diameter. The bending radius of the balloon-shaped section is defined as R. When light transmits through the bending section, the bending radius is so small that a portion of the core mode is coupled into the cladding and then re-coupled back into the core, creating an interferometer due to the differences in effective refractive indices (RIs) between the core and cladding modes.

The two bending sensors are made from a standard commercial single-mode fiber, each with a 10 mm polymer coating layer scraped off, and their bending radii are 4 mm. As shown in Figure 2b, a heat-shrinkable tube and a small iron bar with similar diameters are used to fix the bent sensor through the pressure between the tube and the bar. Figure 2b,c illustrate the structure of the balloon-like bent fiber sensor and a photograph of the real product.

**Figure 3:**
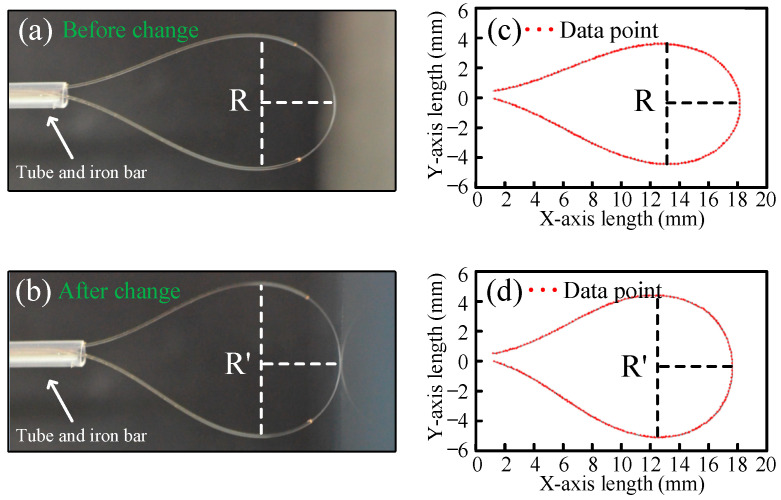
The schematic diagram of the radius variation of the displacement sensor. (**a**) No displacement. (**b**) After displacement. (**c**) Data point for curvature radius fitting without displacement. (**d**) Data point for radius curvature fitting after displacement. R, the initial radius; R’, the radius after the change.

The bending segment induces the coupling between the core mode and the cladding modes [7]. For simplicity, the cladding modes can be grouped together, and the transmission of the balloon-like structure can be considered as a two-beam Mach–Zehnder interferometer (MZI), whose output intensity can be written as follows:

(1) $$I_{out}=I_{co}+I_{cl}+2\sqrt{I_{co}I_{cl}}\cos\left(\Delta \varphi \right)$$

where $I_{co}$ and $I_{cl}$ are the light intensities of the core mode and the cladding mode, and Δφ is the phase difference between the two modes. The total phase difference between the core mode and the cladding mode can be expressed as follows:

(2) $$\Delta \varphi =\frac{2\pi L_{eff}}{\lambda }\Delta n_{eff}$$

where $L_{eff}$ is the effective bent length of the core mode and cladding mode, λ is the wavelength in the bent waveguide, $\Delta n_{eff}=\left(n_{co,eff}-n_{cl,eff}\right)$ is the effective RI difference between the core and cladding modes, and $n_{co,eff}$ and $n_{cl,eff}$ are the core and cladding effective RI values. If the phase difference meets the conditions of $\Delta \varphi =\left(2k+1\right)\pi$, where *k* is an integer, an interference dip will occur at the wavelengths given by the following:

(3) $$\lambda_{dip}=\frac{2L_{eff}\Delta n_{eff}}{2k+1}$$

where $\lambda_{dip}$ is the wavelength of the interference dip. The free spectrum range (FSR) of the transmission spectrum can be approximately written as the following expression:

(4) $$FSR=\frac{\lambda_{dip}^{2}}{\sum_{i=1}\left(dL_{i}\times \Delta n_{i}(R)\right)}$$

where $dL_{i}$ is the differential form of the bent length, R is the radius of curvature, $\Delta n_{i}$ is the difference between the effective refractive index of two main modes, and $\Delta n_{i}$ is related to the bent radius. When the sensor is used to measure the displacement between two objects, the bending sensor is fixed on one of the objects and is pushed against the other object. As illustrated in Figure 3a,b, the bending radius of the balloon-shaped section is changed, resulting in a shift of the interference dip.

When the wavelength of the displacement sensor shifts, it can be mapped to time-domain variations by combining the scanning speed parameters of the swept light source. The relationship between time and wavelength difference can be expressed as follows:

(5) $$T=\frac{\left|\lambda_{ref}-\lambda_{sen}\right|}{v}$$

where $\lambda_{ref}$ and $\lambda_{sen}$ represent the characteristic wavelengths of the reference sensor and the displacement sensor, respectively, and ν denotes the scanning speed of the swept light source. Then, the displacement–time sensitivity of the sensing system can be expressed as follows:

(6) $$S=\frac{\Delta T}{\Delta L}$$

where ΔT represent the change in time difference after displacement is applied to the sensing sensor, and ΔL denotes the value of the displacement change. In this way, the wavelength change is successfully mapped to the time-domain variation. According to actual application scenarios and measurement requirements, the sensing system can adjust its sensitivity and scanning speed by replacing the WSL light source with different scanning speeds.

**Figure 4:**
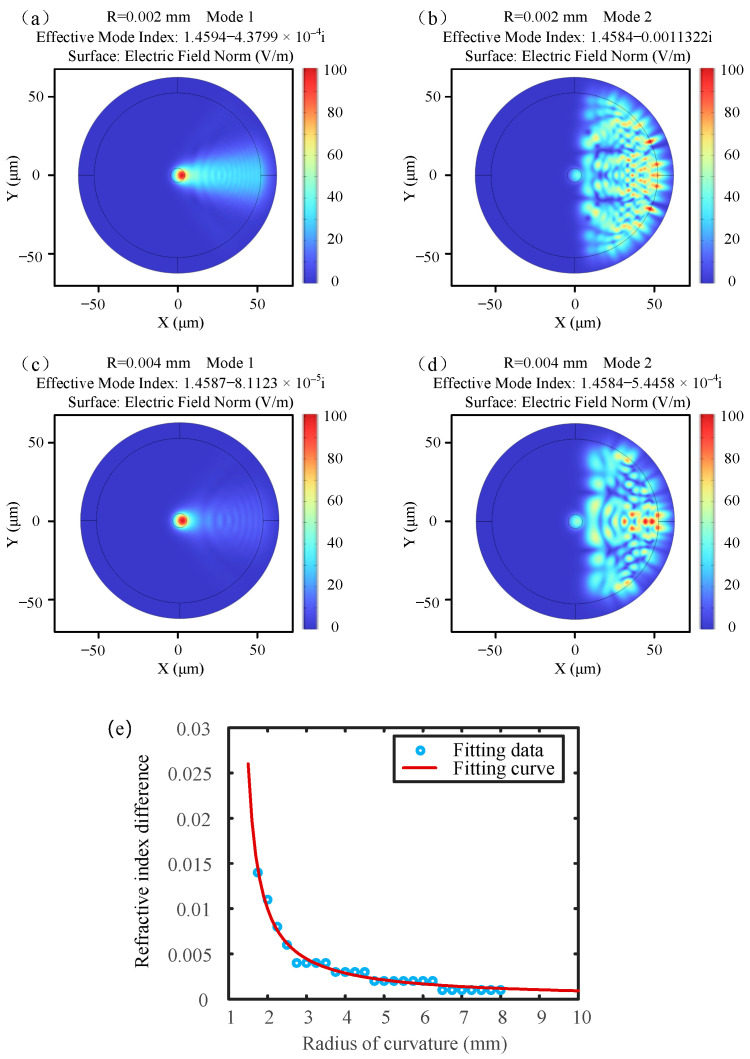
Analysis of the effective refractive index for different curvature radii. (**a**) Mode 1 at a radius of 2 mm; (**b**) Mode 2 at a radius of 2 mm; (**c**) Mode 1 at a radius of 4 mm; (**d**) Mode 2 at a radius of 4 mm; (**e**) Fitting curve of the refractive index difference as a function of the radius.

The mode analysis of single-mode fibers with different bending radii was performed using COMSOL6.2 software. The effective refractive index of the core and the cladding were set to 1.461 and 1.456. Figure 4 shows the electric field distributions of the two main modes at different bending radii, as well as the fitting curve of the refractive index difference as a function of the radius in Figure 4e. It can be observed that as the bending radius increases, the refractive index difference decreases significantly. When the radius exceeds 8 mm, the refractive index difference approaches zero. To further investigate the curvature variation of the displacement sensor, we performed modeling and calculation of the sensor’s curvature radius both without displacement and under displacement in Figure 3c,d. We then substituted the refractive index differences for different curvature radii into Equation (4) to calculate the results. The results indicate that for curvature radii smaller than 8 mm, the total variation in curvature decreases, while the corresponding total refractive index difference increases. It was observed that as the displacement increases, the radius of the bending sensor appears to increase, while the wavelength shifts toward the blue spectrum.

**Figure 5:**
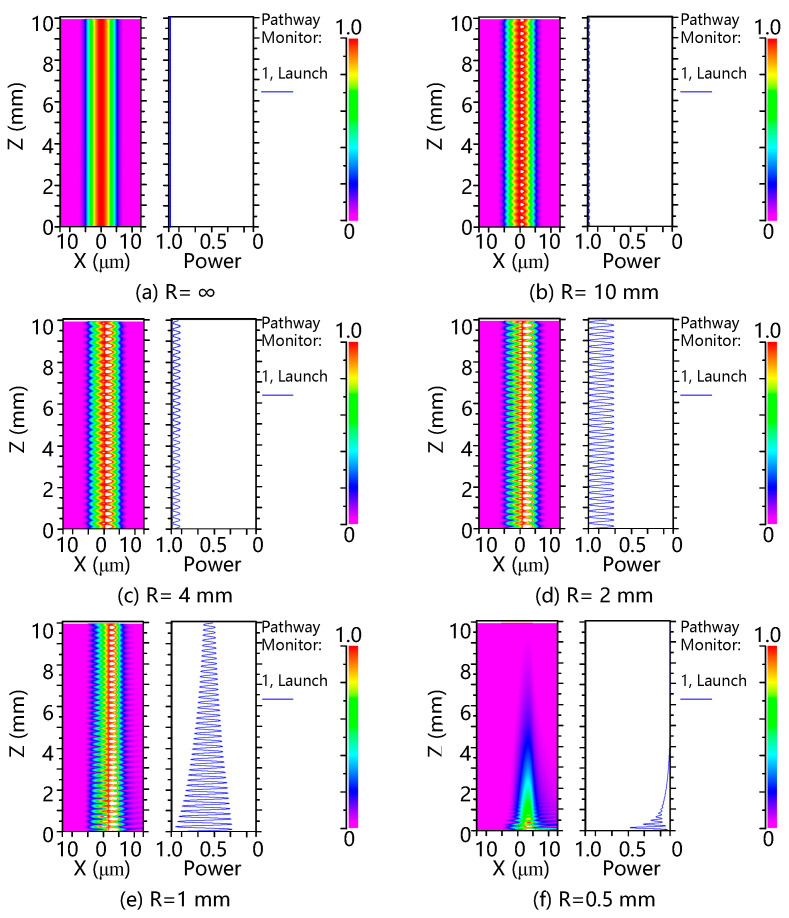
Waveguide simulation of different bending radii. (**a**) R = ∞; (**b**) R = 10 mm; (**c**) R = 4 mm; (**d**) R = 2 mm; (**e**) R = 1 mm; (**f**) R = 0.5 mm.

Figure 5 illustrates the simulation results of the two-dimensional (2D) bent waveguide in the fiber, utilizing the R-soft2018 software based on the beam-propagation method (BPM). A 10 mm length of single-mode fibers under different bending radii was simulated. The effective refractive indices of the core and the cladding were set to 1.461 and 1.456, the diameter of the fiber core was 9 μm, and the incident wavelength was set to 1550 nm. The simulation results in Figure 5a,b show that as the bending radius is greater than 10 mm, no interference pattern appears because little to no light energy in the core waveguide is coupled to the cladding. As the bending radius decreases from 10 mm to 1 mm, as shown in Figure 5c–e, the coupling efficiency between the core mode and cladding mode increases significantly. However, when the radius is smaller than 1 mm in Figure 5f, a certain amount of light energy from the cladding is emitted into the air, leading to a noticeable power loss. Based on the results of the simulations, a high interference spectrum visibility can be obtained when the bending radius is between 2 mm and 4 mm. In the experiment, the selection of a 4 mm bending radius was mainly based on considerations of the fragility of the fiber, the repeatability of sensor fabrication, and convenience. Theoretically, although a smaller bending radius increases sensitivity, it also leads to higher losses, reduced stability, and a higher risk of breakage. Therefore, trade-offs must be made based on actual circumstances.

Moreover, when the environmental temperature changes, the interference dips of both the reference sensor and the displacement sensor will shift in the same direction with similar sensitivity. In the WSL sensing system, the time difference between the two wavelengths corresponding to the interference dips is used to demodulate the displacement. This approach significantly reduces the cross-sensitivity of the temperature effect on displacement measurement. Compared to demodulation systems that rely on a single sensor or different cascaded sensors, this design reduces the impact of environmental temperature on the measurement of other physical parameters.

## 3. Experiment

**Figure 6:**
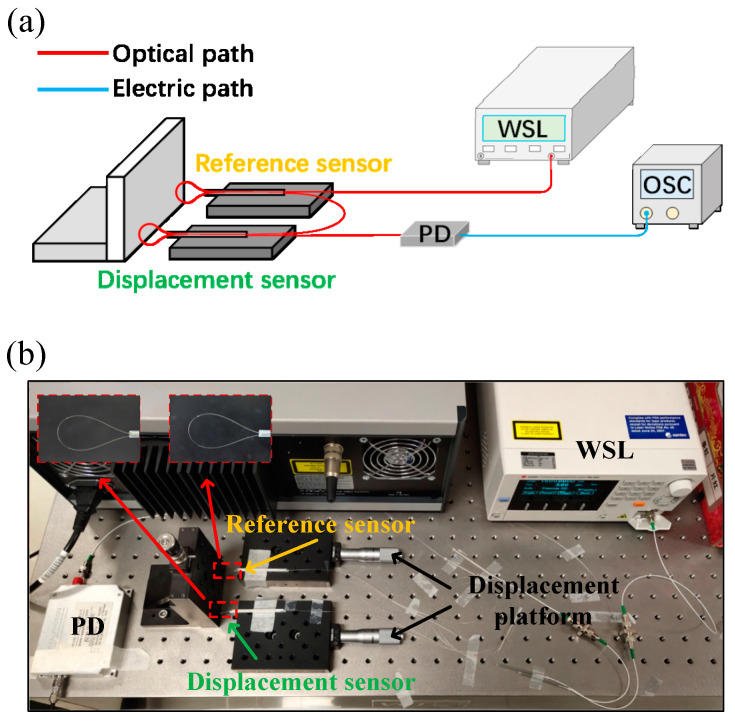
The displacement sensing system based on the WSL and the cascaded balloon-like bent fiber sensor. (**a**) Schematic diagram of the WSL sensing system. (**b**) Photograph of the WSL sensing system. WSL, wavelength-swept laser; PD, photodetector; OSC, oscilloscope.

Figure 6a illustrates the schematic of the WSL sensing system used in the experiment, and Figure 6b shows a photograph of the displacement measurement system in operation. The light source (TSL-550A, Santec, Yokohama, Japan) in our experiment has a sweeping range from 1500 nm to 1630 nm and a precision of 15 pm. The scanning impulses of different wavelengths pass through the reference sensor and the displacement sensor to obtain the reference signal for the subsequent displacement testing. The bending sensor, fixed on a one-dimensional displacement platform, is pushed forward slowly with a constant step size of 10 μm. The balloon-shaped section compresses against the wall as it moves, increasing the bent radius. To ensure the stability of the reference sensor, we will also apply appropriate compression between the reference sensor and the baffle, thereby adjusting and securing the characteristic wavelength position of the reference sensor. After that, the photodetector (PD-50-S-FA-V, Realphoton, Shenzhen, China) with a bandwidth of 20 GHz receives the light power at different wavelengths for time-domain analysis. Finally, waveform data is collected using an oscilloscope (ADS1302CE, Atten, Shenzhen, China), and the time difference between the signals from the reference sensor and the displacement sensor is analyzed to demodulate the sensing parameter.

**Figure 7:**
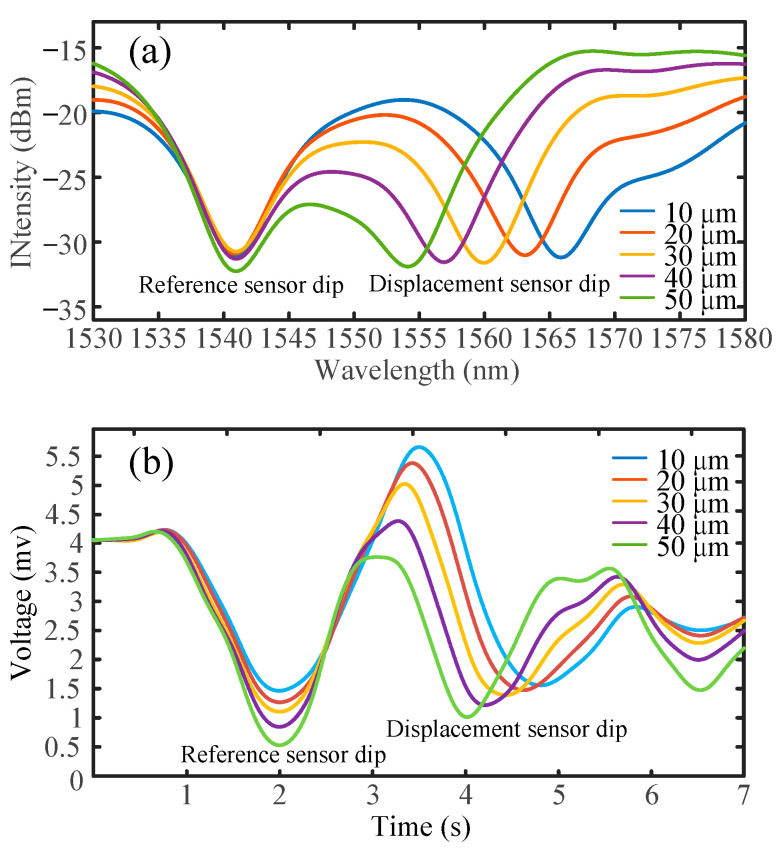
The optical spectra and time waveforms of the reference sensor and the displacement sensor within a range of 10 μm to 50 μm. (**a**) Wavelength intensity data from the optical spectrum analyzer (**b**) Voltage curve data from the oscilloscope.

**Figure 8:**
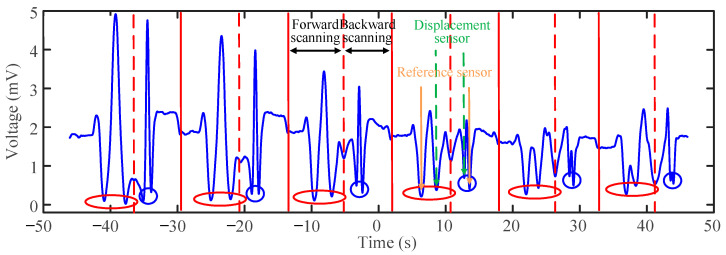
The measured waveform on the oscilloscope with a 20 μm step in a continuous 50 s sampling.

Figure 7a shows the optical spectrum of the reference sensor and the displacement sensor tested at a displacement step of 10 μm. It is evident that the reference sensor dip remains stable, while the displacement sensor dip consistently shifts towards the reference dip at regular intervals. Figure 7b illustrates the time–voltage relationship on the oscilloscope at a forward sweeping speed of 20 nm/s, with a sweeping range from 1500 nm to 1600 nm. Additionally, the sweeping range in our WSL displacement sensing system spans from 1500 nm to 1600 nm and then returns to the starting point (1600 nm to 1500 nm) within one period. Notably, the backward sweep is spontaneous for the WSL system used in the experiment. As a result, the speed of the backward sweep is fixed and cannot be reconfigured. Moreover, the backward sweeping speed is observed to be faster than that of the forward sweep, while the sweeping range remains unchanged. The backward sweeping data are utilized to demodulate the displacement sensing information, as shown in Figure 8.

**Figure 9:**
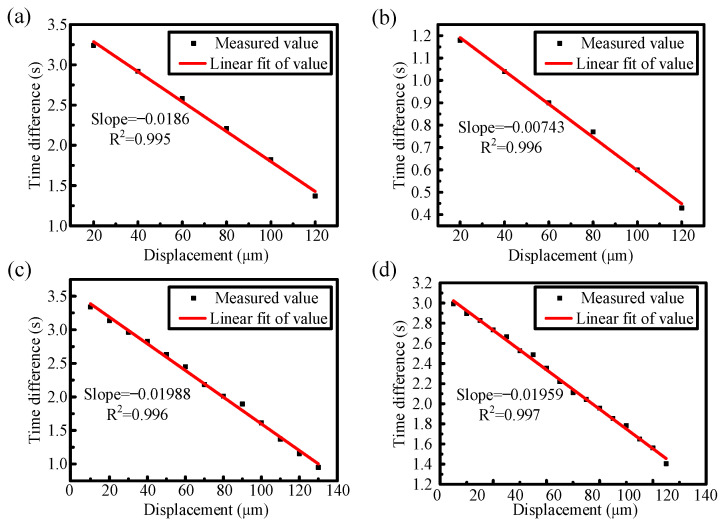
Linear fitting results of displacement measurements under different scanning processes. (**a**) Forward scanning process with a step size of 20 μm (**b**) Backward scanning process with a step size of 20 μm. (**c**) Forward scanning process with a step size of 10 μm. (**d**) Forward scanning process with a step size of 5 μm.

We utilize the WSL system to measure the displacement variations with a step size of 20 μm. The measurement is performed by adjusting the displacement of the platform during the scanning gap time of the WSL. Figure 8 shows the waveform that appeared on the oscilloscope when the step size of 20 μm was continuously measured over six periods, with a displacement range from 20 to 120 μm. Each period consists of two measurement processes: one for forward scanning and one for backward scanning. This configuration enables data collection twice within a single sweeping period, providing enhanced analysis compared to OSA-based demodulation methods. In Figure 8, the solid red line marks the end of each period, while the dashed red line indicates the division between positive and negative scanning. The waveform dips generated by the reference sensors remain constant and serve as reference points for data analysis. The two waveform dips generated by the displacement sensors in the middle gradually shift towards each reference dip as the displacement platform moves. The forward scanning sensing dips are enclosed within the red elliptical curve, while the reverse scanning sensing dips are enclosed within the blue circular curve. From Figure 8, it is observed that as the displacement increases, the interval between the dips gradually decreases. The oscilloscope is used to collect these data for analysis and processing, ultimately creating the displacement–time fitting diagrams shown in Figure 9a,b.

Figure 9 illustrates the relationship between displacement and time difference for both forward and backward scanning. The black solid points represent the time differences observed after displacement changes in the experiment, while the red solid line represents the linear fit of these values. Experimental results demonstrate a strong linear correlation between displacement and time difference. In Figure 9a, the forward sweeping results show a displacement sensitivity of 18.6 ms/μm with a high coefficient of determination (R^2^ = 0.995). Similarly, Figure 9b depicts the backward sweeping results, revealing a sensitivity of 7.43 ms/μm (R^2^ = 0.996). These results indicate that our WSL sensing system can accurately demodulate the micro-displacement variations in a continuous measurement process. Both forward and backward scanning methods are effective for sensing tests, meeting the demands for a simple, single-link, double-sampling-rate, and high-sensitivity micro-displacement sensing system. Furthermore, since the entire system is based on WSL sensing demodulation, it also offers the additional advantages of high resolution and high-speed performance.

Figure 9c shows the testing results of displacement measurement with a step size of 10 μm and a range from 10 to 130 μm. The sweeping speed and wavelength sweeping range are the same as the previous tests. The result is similar to that obtained with a step size of 20 μm, exhibiting a similar sensitivity and a good linear fit. The highest micro-displacement sensitivity is 19.88 ms/μm (R^2^ = 0.996). To verify whether the system can measure smaller displacement variations, we conducted an experiment with a step size of 5 μm, ranging from 5 to 85 μm. As shown in Figure 9d, the sensitivity of the displacement test is 19.59 ms/μm (R^2^ = 0.997), consistent with the other tests. These findings demonstrate that our WSL sensing system can accurately resolve displacements as small as 5 μm. These results confirm that the sensor system can effectively distinguish tiny displacements, even with smaller step sizes.

**Figure 10:**
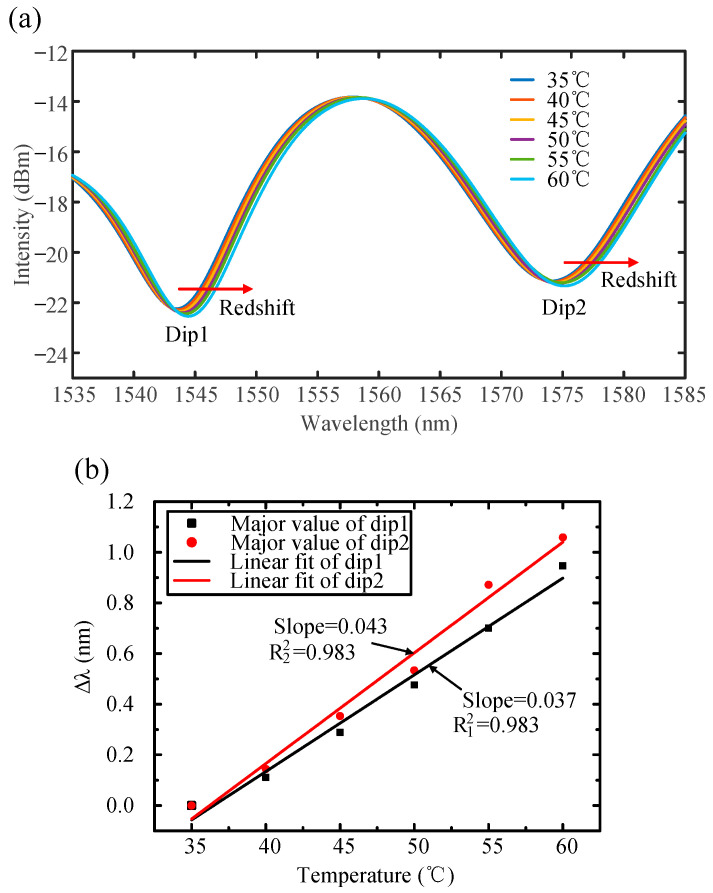
The temperature effect on the displacement sensing system. (**a**) Transmission spectrum of the cascaded balloon-like bending sensor from 35 °C to 60 °C. (**b**) Linear fit of Dip1 and Dip2.

Figure 10 illustrates the effect of temperature on the displacement sensing system. Figure 10a shows the transmission spectrum of the cascaded balloon-like bending sensor from 35 °C to 60 °C. As the temperature increases, the two dips exhibit a redshift. The relationship between the change in wavelength Δλ and temperature variation is shown in Figure 10b. The temperature sensitivity of Dip1 and Dip2 is 38 pm/°C (R^2^ = 0.983) and 43 pm/°C (R^2^ = 0.983), respectively. When measurements are performed using the scanning light source, the actual temperature influence is represented by the difference in wavelength between the two dips. After data analysis, the final temperature sensitivity is reduced to 5 pm/°C and the low cross-sensitivity effect on the displacement sensing system is only 0.25 ms/°C at a sweeping speed of 20 nm/s. This indicates that the system effectively minimizes errors caused by temperature influence in the displacement measurement. If the temperature increases further, materials with a lower thermal expansion coefficient can be chosen for encapsulation to improve the stability of sensor.

Table 1 lists the sensitivity of several displacement sensors based on the traditional fiber sensor demodulation method that utilizes an optical spectrum analyzer. Compared to the displacement and temperature sensitivities of the fiber Bragg grating sensor, the hybrid optical fiber structure sensor, and other bent fiber sensors, our proposed WSL sensing system demonstrates the highest displacement sensitivity and the lowest temperature cross-sensitivity. A micro-displacement sensitivity of 19.88 ms/μm (397.6 pm/μm) is achieved with a low influence of 0.25 ms/°C (5 pm/°C) at a sweeping speed of 20 nm/s, which is not only higher than the displacement sensitivity of the other similar works but also meets the demand of continuous and high-speed demodulation with a low cross-sensitivity.

Other factors influencing the experimental results include environmental conditions and the precision of the measurement instruments. Changes in environmental parameters mainly involve temperature, vibration, and air humidity, while errors caused by instrument precision primarily include the frequency stability of the laser, the accuracy of the displacement platform, and the fabrication errors of the fiber sensor. Therefore, appropriate encapsulation and compensation based on actual conditions will be required during later practical application stages.

## 4. Conclusions

In conclusion, a low cross-sensitivity and non-electric trigger sensing system based on the WSL demodulation method is proposed through a cascaded balloon-like bent fiber sensor and applied to high-sensitivity displacement sensing with a continuous sampling feature for experimental validation. The electric trigger signal is replaced by the dip generated by the light reference sensor, simplifying the complexity of the entire WSL displacement sensing system. The displacement data is obtained by analyzing the time difference between the two dips of the bent fiber sensors, caused by the coupling length variation between the core and the cladding mode. The high displacement sensitivity of our WSL sensing system is 19.88 ms/μm (397.6 pm/μm) in the forward scanning process and 7.43 ms/μm in the backward scanning process with a temperature cross-sensitivity of 0.25 ms/°C (5 pm/°C) over the range of 35 °C–60 °C, which is not only higher than the displacement sensitivity of the other similar works but also meets the demand of continuous and high-speed demodulation with a low cross-sensitivity. Considering the high sensitivity of the sensor to external interference, appropriate encapsulation and compensation based on actual conditions will be required during later practical application stages. Unwanted higher-order modes may also arise during the sensor fabrication process. Therefore, strict control of production parameters will be essential in future commercialization to ensure stability and yield rate. This sensing method offers the advantages of simple configuration, high sensitivity, low cross-sensitivity, and low cost for micro-displacement measurement. In the future, the potential application scenarios for this sensor system include seawater salinity and temperature monitoring, structural health management of buildings, and stability measurement of aerospace components.

## Figures and Tables

**Table 1:** Comparison of several displacement sensing systems in terms of their temperature sensitivity and high-speed measurement potential.

Sensing Structure	High-Speed Measurement	Displacement Sensitivity	Temperature Sensitivity	Ref.
BFS cascaded LPG	No	−306 pm/μm	42.9 pm/°C	[27]
FBG	No	0.0924 pm/μm	113 pm/°C	[28]
BFS cascaded FBG	No	−180 pm/μm	105 pm/°C	[29]
SMF-TCF-SMF	No	15.35 pm/μm	9.42 pm/°C	[30]
BFS	No	71.49 pm/μm	—	[5]
SMF-Capillary-SMF BFS	No	1.68 pm/μm	28.6 pm/°C	[6]
Core-offset BFS	No	306 pm/μm	165 pm/°C	[31]
Double BFS	Yes	19.88 ms/μm (397.6 pm/μm)	0.25 ms/°C (5 pm/°C)	This work

(BFS, bent fiber sensor; LPG, long period grating; FBG, fiber Bragg grating; SMF, single mode fiber; TCF, thin-core fiber).

## Data Availability

Data are contained within the article.
